# Disorders of Gut Microbiota and Plasma Metabolic Profiles May Be Associated with Lymph Node Tuberculosis

**DOI:** 10.3390/microorganisms13071456

**Published:** 2025-06-23

**Authors:** Yun Long, Jiamin Huang, Shasha Zheng, Shimeng Bai, Zhe Liu, Xue Li, Wenying Gao, Xue Ke, Yunyan Tang, Liang Yang, Haijiang Wang, Guobao Li

**Affiliations:** 1 Shenzhen Third People’s Hospital, National Clinical Research Centre for Infectious Disease, The Second Affiliated Hospital of Southern University of Science and Technology, Shenzhen 518112, China; 2 Department of Pharmacology, Joint Laboratory of Guangdong-Hong Kong Universities for Vascular Homeostasis and Diseases, SUSTech Homeostatic Medicine Institute, School of Medicine, Southern University of Science and Technology, Shenzhen 518055, China

**Keywords:** gut microbiota, lymph node tuberculosis, short-chain fatty acid, multi-omics analysis

## Abstract

The association of gut microbiota with lymph node tuberculosis (LNTB) remains unexplored. This study employed metagenomic sequencing and plasma metabolomics analyses to investigate the role of gut microbiota in LNTB patients. Significant alterations in gut microbial diversity were observed in LNTB patients, characterized by a notable reduction in bacterial taxa involved in short-chain fatty acid (SCFA) synthesis, including *Ruminococcus*, *Faecalibacterium*, *Roseburia*, and *Blautia*, compared to healthy individuals. KEGG pathway analysis further revealed that gut dysbiosis could negatively impact SCFA biosynthesis and metabolism. Plasma metabolomics demonstrated disruptions in metabolites associated with SCFA synthesis and inflammation pathways in the LNTB group. Integrated analysis indicated significant correlations between specific gut microbiota (*Blautia*, *Butyricicoccus*, *Coprococcus*, *Ruminococcus*, *Bacteroides*, *Clostridium*) and plasma metabolites, including α-benzylbutyric acid, acetic acid, and succinic acid. Our findings demonstrate that gut microbiota dysbiosis and related metabolic dysfunction significantly reduce SCFA production in LNTB patients, potentially identifying novel therapeutic targets for LNTB management.

## 1. Introduction

Tuberculosis (TB) is one of the leading causes of illness and death worldwide, posing a significant burden on global public health [1]. Caused by Mycobacterium tuberculosis (MTB), TB is a chronic infectious disease that can be categorized into pulmonary TB and extrapulmonary TB. Extrapulmonary TB refers to MTB infections occurring outside the lung parenchyma and can affect almost every organ system. Among these, lymph node tuberculosis (LNTB) is the most common form [2]. LNTB, representing 30% to 55% of extrapulmonary TB cases, is among the most frequent forms of TB outside the lungs [3]. Cervical lymph nodes are the most frequently affected sites. Patients often present with systemic TB symptoms, such as prolonged low-grade fever and night sweats. As the disease progresses, it may lead to complications, including suppurative infections, chronic sinus tract formation, and skin necrosis [4,5]. These conditions are particularly severe in immunocompromised individuals, where the disease can progress rapidly and become life-threatening. Despite the relatively high incidence of LNTB, the molecular mechanisms underlying its pathogenesis remain poorly understood, which has significantly hindered the advancement of effective diagnostic and therapeutic strategies.

The human body hosts a vast population of commensal bacteria, collectively known as the microbiota, which maintain a dynamic interplay with the immune system and play a crucial role in maintaining health. Some studies [6] have demonstrated strong associations between gut microbiota and both infectious and non-infectious diseases. Specifically, alterations in the gut microbial community have been observed in pulmonary tuberculosis (PTB) patients, and dysbiosis of the gut microbiota has been shown to increase host susceptibility to MTB infection. Evidence [7] suggests that MTB infection leads to gut microbiota imbalances, characterized by shifts in the abundance of key bacterial taxa, including reductions in short-chain fatty acid (SCFA)-producing bacteria such as *Ruminococcus* and *Bifidobacterium*. Additionally, gut-derived metabolites, such as indole propionic acid, have been reported to inhibit MTB growth [8].

The advent of omics technologies has provided powerful tools for investigating disease-related disruptions in metabolic homeostasis. These methods have shed light on disease mechanisms across a range of conditions, including cardiovascular disease, cancer disease, and infectious diseases [9,10]. In recent years, the application of omics—particularly metabolomics and lipidomics—in TB research has offered novel insights into host–pathogen interactions and the role of host metabolism in disease progression [11,12]. Several potential clinical biomarkers have been identified for diagnosis and therapeutic monitoring [13,14]. The integration of metabolomics and gut microbiota analysis has emerged as a powerful approach to elucidate disease development, identify critical regulatory factors, uncover associations between the gut microbiome and metabolic products, and explore potential complex molecular networks [15,16,17]. However, the validation of these findings across different TB forms, especially in LNTB, remains insufficient and demands further investigation.

Previous studies on human LNTB have primarily focused on epidemiology, clinical manifestations, pathology, diagnosis, and treatment [18]. However, investigations into disease progression, underlying regulatory mechanisms, and key molecular drivers remain limited. In the present study, we employed a multi-omics strategy combining metagenomic sequencing of fecal samples and untargeted plasma metabolomics to investigate both distinct and shared features in patients with LNTB. Our goal was to identify potential diagnostic biomarkers for LNTB and to provide a comprehensive and in-depth understanding of the molecular mechanisms underlying this disease. By integrating rigorous functional interpretations with multi-omics data, we aimed to uncover microbial and metabolic alterations associated with the pathophysiology of LNTB, ultimately contributing to improved disease management and therapeutic strategies.

## 2. Material and Methods

### 2.1. Clinical Sample Collection

The collection of blood samples, fecal samples, and clinical data was approved by the Ethics Committee of Shenzhen Third People’s Hospital (Approval No. 2025-015). All participants provided informed consent prior to enrollment. The study population was divided into two groups: healthy volunteers (Health group, *n* = 8) and patients with lymph node tuberculosis (LNTB group, *n* = 9). All LNTB cases included in this study were newly diagnosed.

Healthy participants were selected based on stable lifestyles and overall good health. They reported a diverse and balanced diet, regular sleep patterns with adequate nightly rest, and no significant addictive behaviors. None had taken antibiotics within the past year or had a history of substance abuse.

Candidate screening and participant recruitment were conducted strictly according to pre-established criteria [19], which included the following: (i) general physical and health status for the inclusion of healthy volunteers; (ii) diagnostic criteria for TB patients (1); and (iii) age between 18 and 65 years. Exclusion criteria included failure to meet inclusion criteria or the presence of any of the following conditions [19]: prior use of therapeutic medications (including antibiotics), hepatitis, HIV/AIDS, rheumatic or autoimmune diseases; coexisting malignancies; serious infections within the past two weeks; pregnancy or known allergy; breastfeeding or intent to conceive; participation in other clinical trials within the past three months; legally recognized disabilities (e.g., blindness, deafness, mutism, intellectual disability, or physical disability); suspected or confirmed history of alcohol or substance abuse; and any circumstances likely to interfere with follow-up (e.g., frequent job relocation). Participants deemed by investigators to have poor compliance potential were also excluded.

### 2.2. Stool Sample Collection and DNA Extraction

Fresh fecal samples were obtained from both healthy individuals and LNTB patients using sterile collection tubes pre-filled with a stool DNA stabilizing reagent, as provided in the PSP^®^ Spin Stool DNA Plus Kit (Stratec Molecular, Berlin, Germany). Immediately after collection, samples were stored at −20 °C to preserve nucleic acid integrity until further processing. For downstream analysis, approximately 200 mg of each fecal specimen was used for metagenomic DNA extraction, following the standard protocol recommended by the manufacturer.

### 2.3. Metagenomic Sequencing and Data Processing

According to the manufacturer’s instructions (Illumina, San Diego, CA, USA), paired-end sequencing libraries with an average insert size of approximately 350 bp were constructed. Sequencing was performed on the Illumina HiSeq 2500 platform using a paired-end strategy. The raw sequencing data were subsequently processed using the MOCAT2 analysis pipeline [20] to remove low-quality reads, adapter contamination, and human-derived sequences. Specifically, sequencing reads were first trimmed using a minimum length threshold of 30 bp and a quality score cutoff of 20. Adapter sequences were filtered out using an e-value threshold of 0.01, and potential human DNA contamination was eliminated by aligning reads to the human genome with a 90% sequence identity threshold. Following quality control and filtering procedures, each sample yielded approximately 5 GB of high-quality clean data on average. For functional profiling, the high-quality reads were assembled into contigs, followed by gene prediction and clustering against a reference gene catalog. Taxonomic profiling and relative abundance estimation were performed using MetaPhlAn2 [21] with default parameters.

### 2.4. Microbial Diversity and Enterotype Analysis

Alpha diversity, reflecting the microbial richness and evenness within individual samples, was assessed using the Shannon diversity index derived from the species-level taxonomic profiles. To evaluate inter-sample variation in microbial composition (beta diversity), non-metric multidimensional scaling (NMDS) analysis was performed based on the Bray–Curtis distance matrix, calculated from the relative abundance of bacterial genera. This approach allowed for the visualization of overall microbial community structure and differences across sample groups.

### 2.5. Plasma Metabolite Extraction for Untargeted Metabolomics

The extraction of plasma metabolites was performed as follows: 50 μL of plasma from each patient was thawed on ice for approximately 30 min to minimize the degradation of thermolabile metabolites and vortexed gently for 10 s to ensure homogeneity. Then, 150 μL of pre-chilled methanol (−20 °C) containing six internal standards—Acetyl-L-carnitine-(N-methyl-d3), L-phenyl-d5-alanine, L-tryptophan-(indole-d5), leucine enkephalin, SM(d18:1/15:0)-d9, and cholic acid-d5—was added to each sample to monitor extraction efficiency and correct for analytical variation. The mixture was vigorously vortexed for 30 s, followed by centrifugation at 14,000 rcf for 2 min at 4 °C to remove precipitated proteins and debris. Next, 150 μL of the supernatant was transferred to a clean tube and evaporated to dryness under a gentle stream of nitrogen at room temperature. The dried residue was reconstituted in 200 μL of 50% methanol (*v*/*v*), briefly vortexed, and centrifuged again at 14,000 rcf for 2 min at 4 °C. Finally, 100 μL of the clarified supernatant was collected and transferred to autosampler vials for subsequent untargeted metabolomics analysis.

### 2.6. Differential Metabolites Analysis

After statistical analysis, the untargeted LC–MS/MS data were further processed to identify differential metabolites across phenotypic groups. Student’s *t*-test was initially applied to detect statistically significant differences in metabolite levels between groups. The resulting *p*-values were adjusted for multiple comparisons using the Benjamini–Hochberg false discovery rate (FDR) correction to control for type I error. To further investigate the metabolic distinctions among groups, orthogonal partial least squares discriminant analysis (OPLS-DA), a supervised multivariate method, was performed using the metaX software (version 2.0.0) package. This approach enabled the identification of variables contributing most significantly to group separation. Variable importance in projection (VIP) scores were calculated within the OPLS-DA model, and metabolites with VIP scores greater than 1.0 were considered to be the most relevant features for discriminating between groups.

### 2.7. Ethics Statement

All fecal and plasma samples were obtained from the Department of Laboratory Medicine at Shenzhen Third People’s Hospital and were fully anonymized prior to analysis. The study protocol, including the use of these biological specimens, was reviewed and approved by the Institutional Ethics Committee of Shenzhen Third People’s Hospital (Approval No. 2025-015).

### 2.8. Statistical Analysis

Statistical analyses were conducted using GraphPad Prism software (version 9.5.1). Data were analyzed with Mann–Whitney U test, and *p* values were corrected using the Benjamini–Hochberg method. The results represent the means ± SD of three independent experiments unless specified otherwise. *p* values < 0.05 were considered statistically significant. False discovery rate controls were set at 10% (*q* < 0.1) using the Benjamini–Hochberg procedure. Details for all statistical tests can be found in the figure legends.

## 3. Results

### 3.1. The Gut Microbiota Composition Is Significantly Altered in LNTB Patients

In the present study, a total of eight healthy volunteers (Health group) and nine patients with LNTB were enrolled in this study. Relevant clinical data were statistically analyzed in Supplementary Tables S1–S5. Comparative analysis revealed that diagnostic indicators were significantly higher in the LNTB group than in the healthy controls (Tables S1–S5).

**Figure 1:**
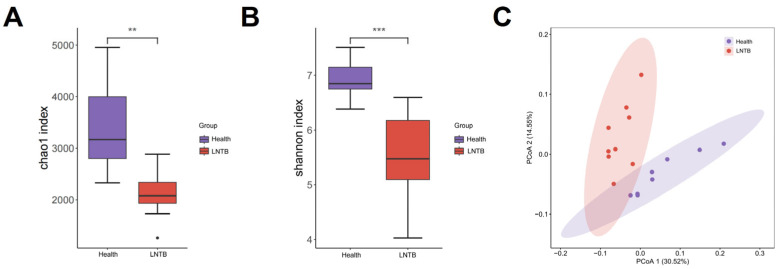
Alpha and beta diversity analyses comparing differences between groups. (**A**,**B**) The Chao1 and Shannon indices were both lower in the treatment group compared to the control group. (**C**) PCoA analysis based on log-transformed metagenomic species abundance. ** *p* < 0.01, *** *p* < 0.001.

To investigate the gut microbiota composition between the Health and LNTB groups, metagenomic sequencing was performed in the present study. In terms of microbial richness and diversity, both the Chao1 and Shannon indices showed a significant reduction in alpha diversity in the LNTB group compared to healthy controls (*p* < 0.01; Figure 1A,B). These findings suggest that the gut microbial community was markedly altered by LNTB, potentially leading to disruptions in microbial homeostasis and decreased microbial diversity. Beta diversity was assessed by calculating the weighted UniFrac phylogenetic distances based on all ASVs. Principal coordinate analysis (PCA) revealed that the differences in community composition between groups were primarily driven by changes in the gut microbiota of LNTB patients (Figure 1C).

These results revealed that the gut microbial composition in LNTB patients was significantly different from that of healthy individuals.

### 3.2. The Composition of Microbiota in LNTB Is Distinct from Healthy Control

**Figure 2:**
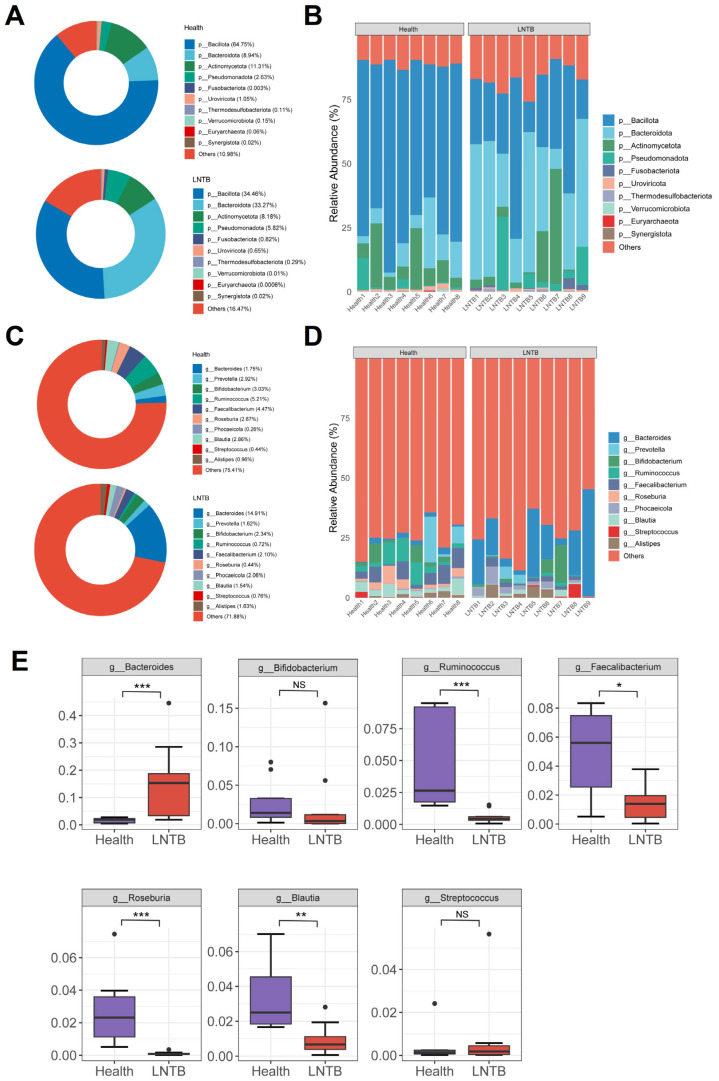
The composition of microbiota in LNTB is distinct from Health. Relative abundance of the most predominant phyla in various sample groups was visualized with (**A**) donut chart and (**B**) column chart. Relative abundance of the most predominant genus in various sample groups was visualized with (**C**) donut chart and (**D**) column chart. (**E**) Analysis of the relative abundance of the most abundant genera associated with SCFA synthesis in LNTB vs. Health. * Indicated the significance for LNTB versus Health. NS: not significant. * *p* < 0.05, ** *p* < 0.01, *** *p* < 0.001.

The human gut microbiota is predominantly composed of the following major phyla: *Bacillota*, *Bacteroidetes*, *Actinobacteria* and *Proteobacteria*, whose abundance dynamics are closely associated with the host’s physiological state [22]. In the present study, the relative abundances of *Bacillota* and *Bacteroidetes* in the healthy group were 64.75% and 8.94%, respectively, while in patients with LNTB, they were 34.46% and 33.27%, respectively (Figure 2A,B). Notably, the abundance of *Actinobacteria* was significantly higher in the LNTB group than that in the healthy group (Figure 2A).

At the genus level, compared with healthy individuals, the LNTB group exhibited varying degrees of dysbiosis involving several genera, including *Bacteroides*, *Prevotella*, *Bifidobacterium*, *Ruminococcus*, *Faecalibacterium*, *Roseburia*, *Phocaeicola*, *Blautia*, *Streptococcus*, and *Alistipes* (Figure 2C,D). Among these genera, *Ruminococcus*, *Faecalibacterium*, *Roseburia*, and *Blautia* were significantly decreased in the LNTB group compared to the healthy group, as illustrated in Figure 2E. The microbial composition at the species level was shown in Figure S1. It is worth noting that these genera are known producers of SCFAs, such as butyrate, which play key roles in maintaining intestinal health and immune function.

### 3.3. Differential Microbial Biomarkers Were Identified with LEfSe

**Figure 3:**
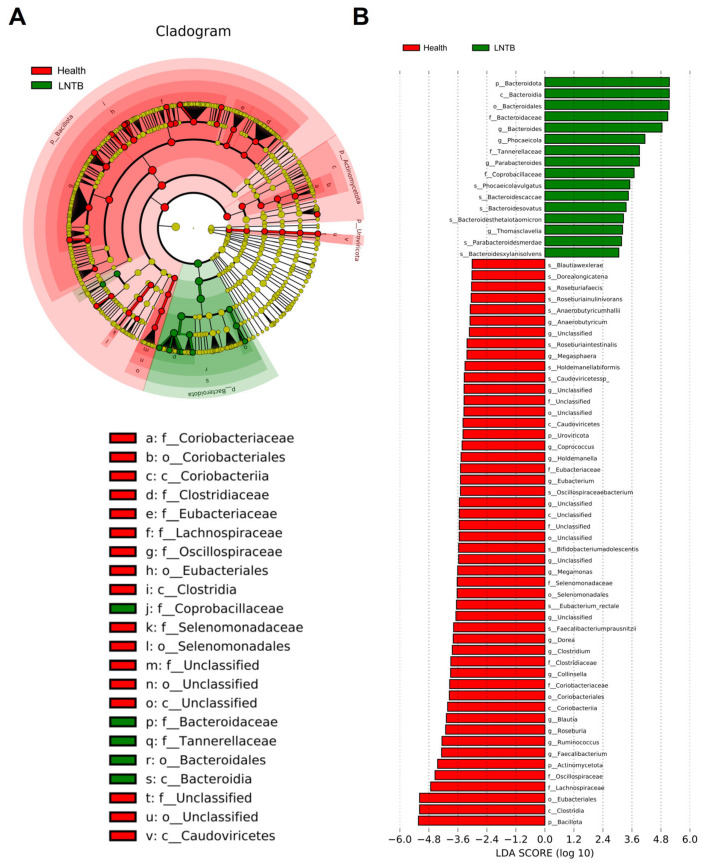
Gut microbiota characteristics in patients with lymph node tuberculosis. (**A**) The phylogenetic tree generated by LEfSe analysis showing the distribution of microbial taxa from the phylum to genus level between the LNTB group and the control group. (**B**) The histogram of LDA scores identifying significantly different bacterial taxa between patients and controls (LDA score > 2.0).

To identify differentially distributed fecal microbiota, we conducted Metastat and linear discriminant analysis (LDA) effect size (LEfSe) analyses. These approaches provide valuable insights into the microbial differences between the LNTB and healthy groups. According to the LDA scores (Figure 3A,B), several taxa were enriched in the healthy group. At the species level, *Blautiawexlerae*, *Faecalibacteriumprausnitzii*, *Eubacterium_rectale*, *Roseburiafaecis*, *Roseburia inulinivorans*, *Anaerobutyricumhallii*, showed significantly higher abundance in healthy individuals. At the genus level, *Faecalibacterium*, *Roseburia*, *Eubacterium*, *Anaerobutyricum*, *Clostridium*, and *Butyricicoccus* were also more abundant in the healthy group. The specific associations between microbial genera and species and different types of SCFAs (including butyrate, acetate, and propionate producers) were illustrated in Figure S2. And the heatmaps of genus- and species-level differential analyses based on fecal metagenomic data from LNTB patients and healthy controls were shown in Figures S3 and S4.

These taxa are well-known key SCFA-producing bacteria, particularly involved in butyrate synthesis, which is closely associated with maintaining colonic health and immune homeostasis. Subsequently, the microbial biomarkers identified by LEfSe would be incorporated into a network analysis to explore associations between gut microbiota and plasma metabolomic profiles.

### 3.4. KEGG Analysis of Gut Microbiota in LNTB Patients and Healthy Individuals

**Figure 4:**
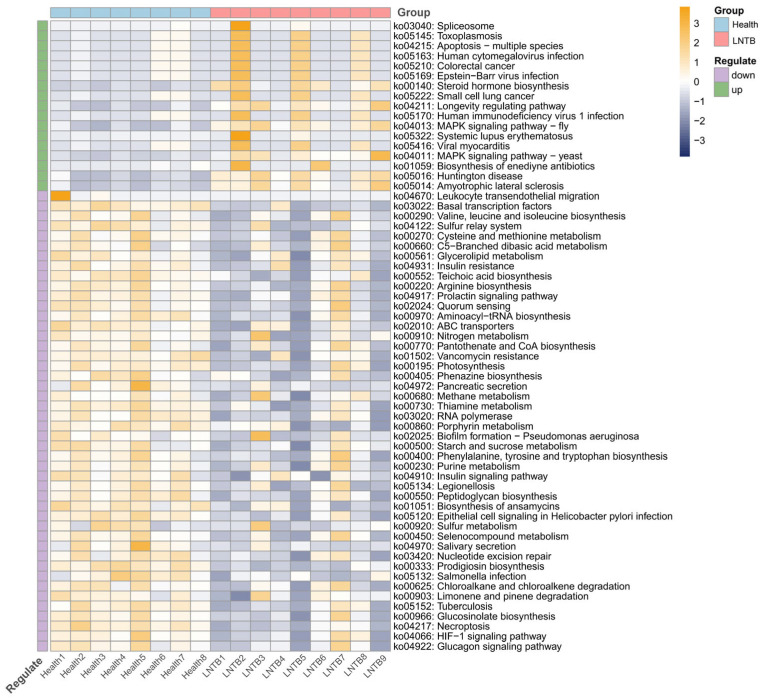
KEGG pathway heatmap of fecal metagenomic data from LNTB patients and healthy controls.

In the present study, functional annotation of the metagenomic microbiota was performed to investigate the potential functions of the gut microbiome and its interactions with the human host. As shown in Figure 4, compared to healthy individuals, several signaling pathways—including those associated with colorectal cancer and small cell lung cancer—were found to be upregulated in the LNTB group. In contrast, pathways related to the biosynthesis of valine, leucine, and isoleucine, as well as the insulin and glucagon signaling pathways, were significantly downregulated.

The biosynthesis of valine, leucine, and isoleucine has been reported to be closely associated with SCFA production, while insulin and glucagon signaling pathways are known to be critical for host metabolic regulation [23,24,25]. SCFAs are considered important metabolic products of the gut microbiota and have been shown to play key physiological roles. These include the maintenance of intestinal mucosal barrier integrity, inhibition of tumor cell proliferation and differentiation, suppression of intestinal inflammation, and stabilization of the gut microenvironment.

Therefore, the observed alterations suggest that SCFA biosynthesis and host metabolic processes may be disrupted in LNTB patients through functional changes in the gut microbiota.

### 3.5. SCFA-Related Metabolic Dysregulation in LNTB

Our results revealed that gut microbiota dysbiosis occurred in the LNTB group. Such compositional alterations provide a foundation for further functional characterization through virulence factor databases (e.g., VFDB) and pathway enrichment analyses. As shown in the VFDB results (Figure S5), the dysbiotic gut microbiota may contribute to immune evasion and other host-related pathological processes.

**Figure 5:**
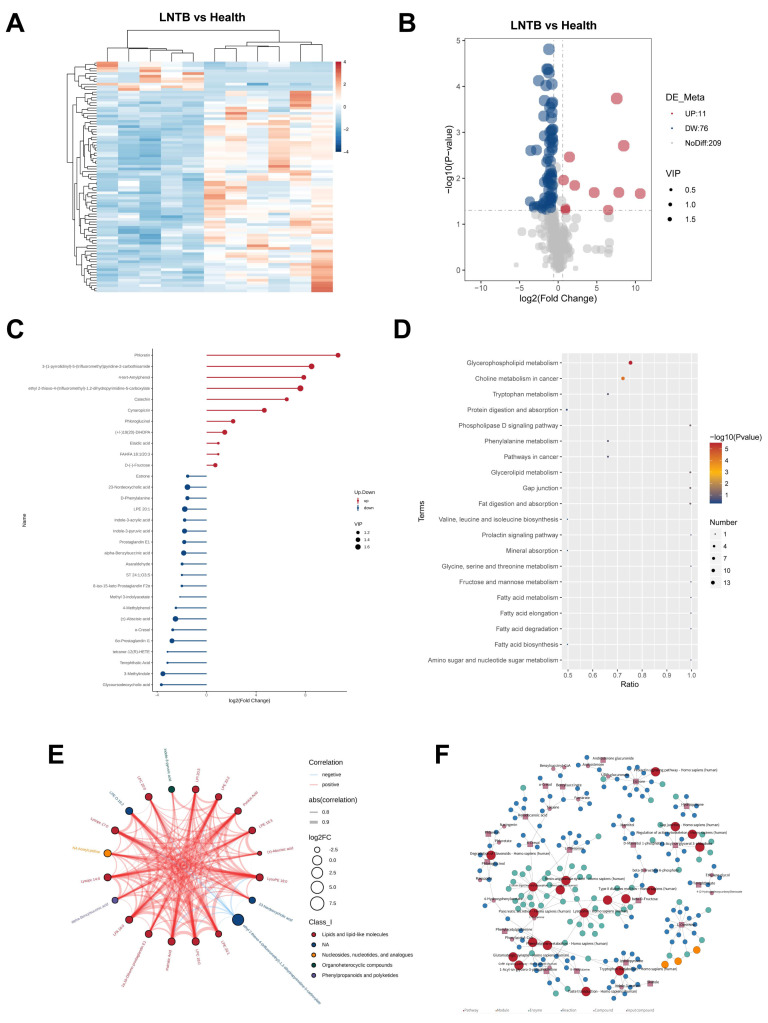
Integrated plasma metabolomics analysis reveals differential metabolic signatures between LNTB patients and healthy controls. (**A**) The relative intensity of differentiated plasma metabolites was depicted by the heatmap between the Health group and LNTB group. (**B**) Volcano plot of differential metabolites. (**C**) Variable importance in projection (VIP) plot from OPLS-DA analysis displaying key differentially metabolites discriminating LNTB from controls. (**D**) KEGG pathway enrichment analysis of differential plasma metabolites. (**E**) The chord diagram illustrating correlation coefficients among differential metabolites. (**F**) KEGG regulatory network illustrating the intersections among metabolic pathways.

To further investigate alterations in the plasma metabolic profiles of LNTB patients compared to healthy individuals, untargeted plasma metabolomics analysis was conducted. As shown in the heatmap of differentially expressed metabolites (Figure 5A), a total of 87 significantly altered metabolites were identified in the negative ion mode in the LNTB group, with 11 metabolites being upregulated and 76 downregulated compared to the healthy controls. The corresponding volcano plot is shown in Figure 5B.

Among these, 30 significantly different metabolites were identified between the LNTB and healthy groups, including D-(–-fructose, α-benzylsuccinic acid, fatty acid-hydroxy fatty acid esters, tetranor-12(R)-HETE, glycochenodeoxycholic acid, and 23-deoxycholic acid, as shown in Figure 5C. These metabolites have been associated with either SCFA synthesis or inflammatory processes.

In addition, pathway enrichment analysis based on the KEGG database revealed a total of 20 significantly altered pathways, as shown in Figure 5D. These pathways included the biosynthesis of valine, leucine, and isoleucine; fructose and mannose metabolism; fatty acid metabolism; choline metabolism in cancer; and other cancer-related signaling pathways. Figure 5E presents a chord diagram illustrating the correlation coefficients among differential metabolites, while Figure 5F displays a KEGG-based regulatory network highlighting the intersections and functional relationships among the affected metabolic pathways.

These findings suggest that SCFA biosynthesis is indeed disrupted in patients with LNTB, and that multiple metabolic pathways involved in energy production and inflammation are affected.

### 3.6. Microbial Signatures Associated with Altered Plasma Metabolites in LNTB Patients

Multi-omics data were utilized to comprehensively characterize the fecal microbiota and plasma metabolite profiles from multiple perspectives. The observed positive or negative correlations between microbial taxa and metabolites may indicate that specific microbial species mediate the production or consumption of corresponding metabolites, or that certain metabolites promote or inhibit the growth of associated microbes.

**Figure 6:**
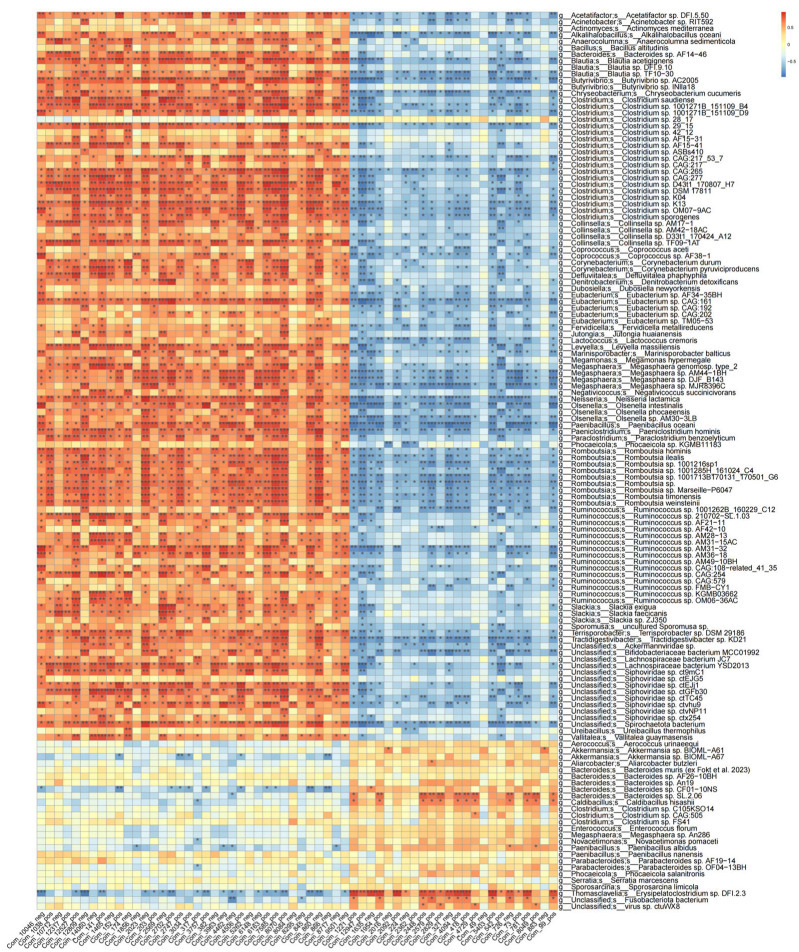
The heatmap illustrates the relationships between gut microbiota and the differential plasma metabolites. Red indicates a positive correlation, while blue indicates a negative correlation. * *p* < 0.05, ** *p* < 0.01, *** *p* < 0.001.

To investigate these associations, large-scale correlation analyses were performed between the differentially abundant fecal genera and plasma metabolites (Figure 6). Using Spearman correlation analysis, the significant associations (*p* < 0.05) were identified in the LNTB and healthy groups.

The correlation analysis between differential fecal genera and plasma metabolites revealed that several taxa, including *Blautia* (species: *Blautia acidifaciens*), *Butyricicoccus* AC2005 (species: AC2005 AF38-1), *Coprococcus* (species: AF38-1), *Ruminococcus*, *Bacteroides*, and *Clostridium* (species: C105K04), were significantly correlated with plasma metabolites, such as α-benzylbutyric acid, 2-({2-[(3-methyl-5-pyridazinyl)amino]-2-oxoethyl}thio)acetic acid, 2-{2-[(1-methyl-1H-pyrazol-5-yl)amino]-2-oxoethoxy}acetic acid, and diethyl-2-(3-chloroanilino)succinic acid.

### 3.7. Functional Pathway–Metabolite Interactions Reveal Microbial Contributions to Host Metabolism in LNTB

**Figure 7:**
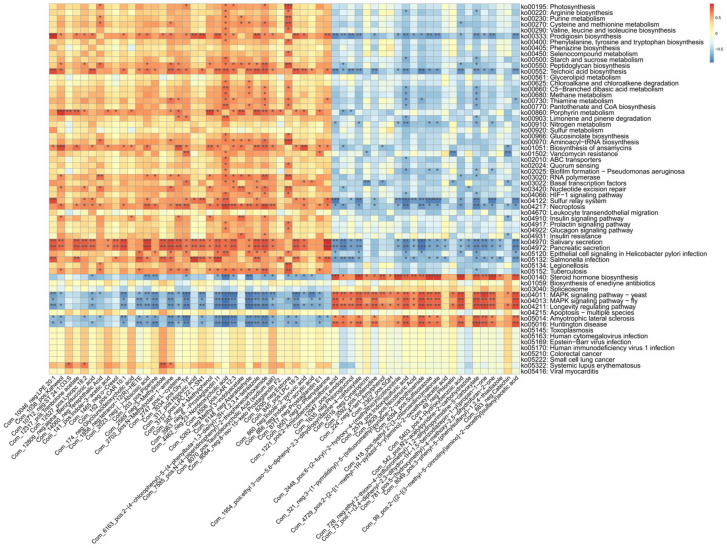
The heatmap illustrates the relationships between KEGG signaling pathways enriched by differential fecal microbiota composition and the differential plasma metabolites. Red indicates a positive correlation, while blue indicates a negative correlation. * *p* < 0.05, ** *p* < 0.01, *** *p* < 0.001.

To further investigate these associations, large-scale correlation analyses were also performed between gut microbiota KEGG pathways and plasma metabolites (Figure 7).

The correlation analysis between gut microbiota KEGG pathways and plasma metabolites revealed that the valine, leucine, and isoleucine biosynthesis pathway was positively correlated with 23-deoxycholic acid and estrone. Additionally, the insulin signaling, glucagon signaling, and diabetes-related pathways also showed positive associations with 23-deoxycholic acid and estrone. Notably, 23-deoxycholic acid has been implicated in the development of diseases, such as colorectal cancer and liver cancer, potentially due to its ability to induce oxidative stress and inflammation within the gut. Meanwhile, the valine, leucine, and isoleucine biosynthesis pathway has been closely associated with the synthesis of SCFAs.

Collectively, these findings highlight the existence of a complex host-microbiota-metabolite interaction network in the context of lymph node tuberculosis.

## 4. Discussion

Lymph node tuberculosis (LNTB) remains a serious threat to human health, and the identification of potential biomarkers, along with the elucidation of underlying pathogenic mechanisms, is of great significance for improving clinical diagnosis and therapeutic interventions. Many research studies [7,26] have demonstrated that alterations in the composition and function of the gut microbiota are closely associated with infectious and immune-related diseases. Therefore, characterizing the taxonomic and functional features of the gut microbiota in LNTB patients, as well as exploring the interactions between gut microbiota and plasma metabolites, is of considerable scientific and clinical value. In the present study, we identified specific gut microbial dysbiosis and plasma metabolic imbalances, and further investigated their functional alterations and interrelationships. These associations underscore the crucial role of host–microbiota–metabolite interactions in the pathogenesis and progression of LNTB.

Until now, multi-omics analyses of the gut microbiota and metabolism in LNTB remain largely unexplored. In the present study, we demonstrated that patients with LNTB exhibit significant alterations in gut microbiota composition compared to healthy individuals. Notably, there was a marked reduction in the relative abundance of SCFA-producing genera, including *Butyricicoccus*, *Faecalibacterium*, *Blautia*, *Roseburia*, and *Ruminococcus*. In addition, metabolomic profiling revealed 87 significantly altered metabolites in the LNTB group compared to controls, of which 11 were upregulated and 76 were downregulated. Previous studies [7] have reported that pulmonary tuberculosis (PTB) and anti-tuberculosis treatment can significantly disrupt the gut microbiota. Compared with healthy controls, *Mycobacterium tuberculosis* (M.tb) infection has been associated with a slight reduction in gut microbial α-diversity, primarily linked to changes in the abundance of *Bacteroidota* [27]. The phylum *Bacteroidota* is the second most dominant bacterial group in the healthy human colon, accounting for approximately 23% of the gut microbiota [28]. Alterations in *Bacteroidota* have also been shown to significantly influence host metabolism and energy homeostasis [29]. In the present study, we similarly observed a modest decrease in α-diversity and alterations in *Bacteroidota* abundance in patients with LNTB compared to healthy individuals. Consistent with previous findings in PTB, our results further support the notion that M.tb infection leads to notable changes in gut microbial diversity and composition, thereby reinforcing the reliability and biological relevance of our observations. Notably, unlike previously reported findings, the proportion of *Bacteroidota* was more substantially increased in the gut microbiota of LNTB patients, suggesting that *Bacteroidota* may be more sensitive to LNTB-associated changes. Several research studies [30,31] have reported a significant reduction in *Bacillota* in patients with tuberculosis, which may be associated with immune dysregulation induced by *Mycobacterium tuberculosis* infection. Consistent with these findings, our study also revealed a marked decrease in the abundance of *Bacillota* in individuals with LNTB.

However, compared with previous studies [32] on pulmonary tuberculosis (PTB), the dysbiosis of butyrate- and propionate-producing genera in the gut microbiota of LNTB patients shows notable differences. Prior research has demonstrated significant alterations in both the taxonomic composition and functional characteristics of the gut microbiota in patients with active TB compared to healthy controls. Specifically, genera, such as *Prevotella* and *Bifidobacterium,* were found to be more abundant in healthy individuals, whereas TB patients exhibited increased levels of SCFA-producing genera, including *Faecalibacterium*, *Roseburia*, *Eubacterium*, and *Phascolarctobacterium*, which are known producers of butyrate and propionate [32]. In contrast, the present study revealed a significant reduction in key butyrate-producing genera in the fecal samples of LNTB patients, including *Butyricicoccus*, *Coprococcus*, *Blautia*, *Roseburia*, and *Ruminococcus*. These findings suggest that SCFA-associated microbial alterations in LNTB may follow a different pattern from those observed in PTB, potentially reflecting distinct host–microbiota interactions in extrapulmonary TB.

Blaak et al. [33] have revealed that SCFAs, including acetate, propionate, and butyrate, are key microbial metabolites derived from the fermentation of indigestible carbohydrates and play a vital role in maintaining gut and metabolic health [34]. SCFAs support intestinal integrity by regulating luminal pH, stimulating mucus production, fueling epithelial cells, and modulating immune responses. Systemically, they influence appetite, energy balance, glucose homeostasis, and immunity. Among them, butyrate exerts strong anti-inflammatory effects and modulates immune signaling via the NF-κB pathway. In active pulmonary tuberculosis, intestinal butyrate levels were found to be fivefold lower than in healthy controls [35], accompanied by reduced abundance of SCFA-producing bacteria and related metabolic pathways [36,37]. This disruption may compromise mucosal defenses and promote pathogenic colonization, contributing to impaired immune function. Consistently, our metabolomic data revealed a dysregulation of SCFA-related metabolites in the plasma of patients with LNTB. Notably, metabolomic analysis in the present study revealed abnormal levels of several metabolites in the peripheral blood of patients with LNTB, including D-(-)-fructose, α-benzylsuccinic acid, fatty acid-hydroxy fatty acid esters, tetranor-12(R)-HETE, glycoursodeoxycholic acid, and 23-deoxycholic acid-metabolites, that are closely associated with SCFA biosynthesis. In addition, several key metabolic pathways were found to be suppressed in LNTB patients, including valine, leucine, and isoleucine biosynthesis, fructose and mannose metabolism, and fatty acid metabolism pathways. These alterations further support the notion that SCFA biosynthesis is disrupted in LNTB. Moreover, integrative metagenomic and metabolomic analyses indicated that the biosynthesis pathway of branched-chain amino acids (valine, leucine, and isoleucine) was positively correlated with the production of 23-deoxycholic acid and estrone. Significant changes were also observed in the abundance of SCFA-associated bacterial genera, including *Blautia* (acetate-producing), *Butyricimonas* (AC2005), *Faecalicoccus* (AF38-1), *Ruminococcus*, and *Clostridium* sp. C105K04. Dysregulation of these taxa was closely linked to the observed alterations in SCFA-related metabolites, further illustrating the disruption of SCFA metabolism and its association with gut microbiota imbalance in LNTB patients. These findings suggest that dysbiosis of specific SCFA-associated microbial populations may influence key physiological processes, such as appetite regulation, energy expenditure, glucose homeostasis, and immune modulation, in individuals with LNTB. This highlights the potential importance of targeting the gut microbiota and SCFA metabolism in the clinical management and therapeutic strategies for LNTB.

Previous studies [37] have reported that *Clostridium* species play an important role in maintaining intestinal homeostasis, protecting the mucosal barrier, and promoting host metabolism. These bacteria are capable of producing SCFAs through the fermentation of dietary fibers. During anti-tuberculosis treatment, the gut microbiota is involved in the metabolic processing of drugs. As treatment progresses and host immunity improves, the abundance of *Clostridium* has been observed to increase significantly, coinciding with clinical improvement in tuberculosis patients. *Prevotella*, a genus whose primary fermentation products include lactic acid, isovaleric acid, and acetic acid, has also been implicated in SCFA metabolism. Among these, acetate is the most abundant SCFA in the peripheral circulation. Therefore, a reduction in *Prevotella* may lead to decreased acetate production, contributing to an overall reduction in SCFA levels [32]. LNTB remains a challenging condition to treat, often requiring prolonged therapeutic regimens. In this study, we report for the first time that several butyrate-producing species—including *Faecalibacterium prausnitzii*, *Butyricicoccus pullicaecorum*, and *Eubacterium* rectale—were significantly more abundant in healthy controls compared to LNTB patients. At the genus level, SCFA-associated taxa, such as *Butyricicoccus*, *Faecalicoccus*, *Blautia*, *Roseburia*, and *Ruminococcus,* were also found in higher abundance in healthy individuals. These findings suggest that SCFA-producing bacterial populations may play a beneficial role in modulating host immunity and could potentially serve as targets for future microbiota-based therapeutic strategies for LNTB. Additionally, these microbial taxa may hold promise as non-invasive biomarkers for the diagnosis or monitoring of lymph node tuberculosis.

We acknowledge that further research is needed to address several limitations of the present study. First, although we are the first to characterize gut microbiota dysbiosis in patients with LNTB in our present acknowledgment, the diagnostic performance of the identified combined biomarker panel requires independent validation in larger and geographically diverse cohorts. This is essential to prevent potential overestimation of diagnostic accuracy due to the limited sample size and single-center design of our study. Moreover, given the current scarcity of related studies, many of the observed associations require more robust confirmation through expanded sample collections and experimental validation, including in vivo animal models. According to previous studies [38,39,40], antibiotics—especially broad-spectrum antibiotics—can eliminate a large number of gut microorganisms, including beneficial bacteria, leading to a significant reduction in species richness and diversity of the gut microbiota. To avoid this confounding effect, the present study only included patients who were newly diagnosed with lymph node tuberculosis and had not received any prior medication. Although this strict inclusion criterion limited the number of samples collected, the low heterogeneity among the included samples ensured their validity. In future studies, we plan to expand the sample size to enhance the robustness of our findings. Moreover, while gut microbiota holds promise as a potential diagnostic tool for LNTB, microbiota-based diagnostic approaches must undergo further large-scale investigations to evaluate their accuracy and specificity before clinical implementation can be considered.

## 5. Conclusions

The present study highlights a potential link between gut microbiome dysbiosis and altered plasma metabolites in patients with LNTB. By integrating metagenomic sequencing with untargeted metabolomic profiling, significant disruptions in both gut microbial composition and plasma metabolic signatures were identified in LNTB patients. These findings offer valuable insights into potential diagnostic biomarkers for extrapulmonary tuberculosis. Specifically, bacterial genera and species associated with SCFA production were notably depleted in LNTB patients. Concurrently, significant alterations in SCFA-related plasma metabolites indicated impaired SCFA synthesis and disruptions in energy metabolism and inflammatory pathways. Furthermore, correlation analyses demonstrated strong associations between gut microbial changes and key metabolic pathways. Overall, this study enhances our understanding of the complex interactions between the gut microbiome, metabolic disturbances, and tuberculosis pathophysiology, especially in extrapulmonary forms and comorbidities, such as diabetes.

## Data Availability

The raw research data have been submitted to the NCBI via the Sequence Read Archive (SRA) and can be accessed at the following link: https://www.ncbi.nlm.nih.gov/sra/PRJNA1273449 (Accession Number: PRJNA1273449).

## Supplementary Materials

The following supporting information can be downloaded at: https://www.mdpi.com/article/10.3390/microorganisms13071456/s1, Figure S1: Species-level microbial composition in Health and LNTB groups. Figure S2: Network diagram of SCFA-producing microbes at the genus and species levels. Figure S3: Heatmap of genus-level differential analysis based on fecal metagenomic data from LNTB patients and healthy controls. Figure S4: Heatmap of species-level differential analysis based on fecal metagenomic data from LNTB patients and healthy controls. Figure S5: Heatmap of virulence factor profiles at Level 2 based on VFDB annotations from fecal metagenomic data of LNTB patients and healthy controls. Table S1: Basic Information of LNTB patients. Table S2: Diagnosis and Comorbidities of LNTB patients. Table S3: Lymph Node Specimen Pathological Examination Items. Table S4: Blood Specimen Laboratory Tests of LNTB patients. Table S5: Lymph Node Specimen Examination Items of LNTB patients. Table S6: The list of correlation-associated metabolites.
