# Supplementary material for: Disorders of Gut Microbiota and Plasma Metabolic Profiles May Be Associated with Lymph Node Tuberculosis
Source: Microorganisms. 2025 Jun 23;13(7):1456. doi: 10.3390/microorganisms13071456 (PMC12298930; doi:10.3390/microorganisms13071456)
Supplement: Supplementary file 1 [file microorganisms-13-01456-s001.zip › microorganisms-3653625-supplementary.pdf]

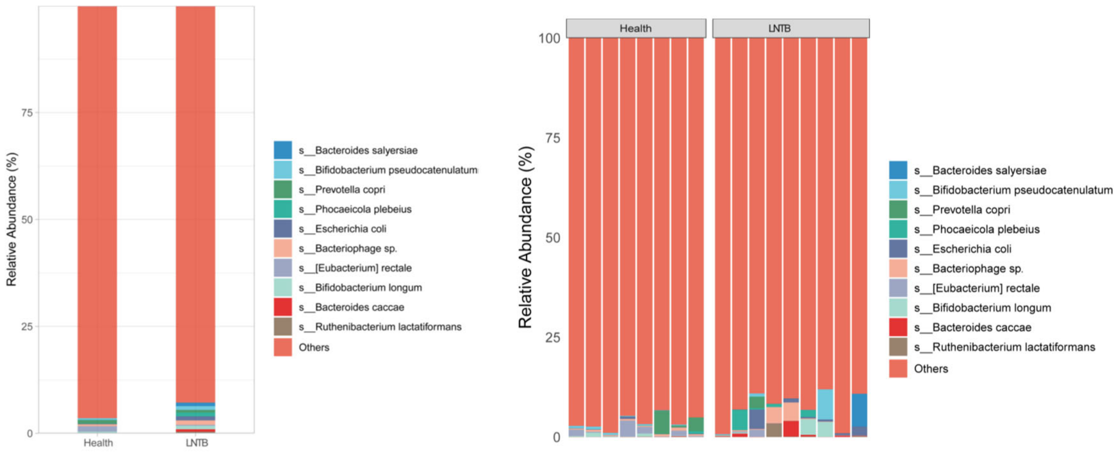

Figure S1. Species-level microbial composition in control and treatment groups.

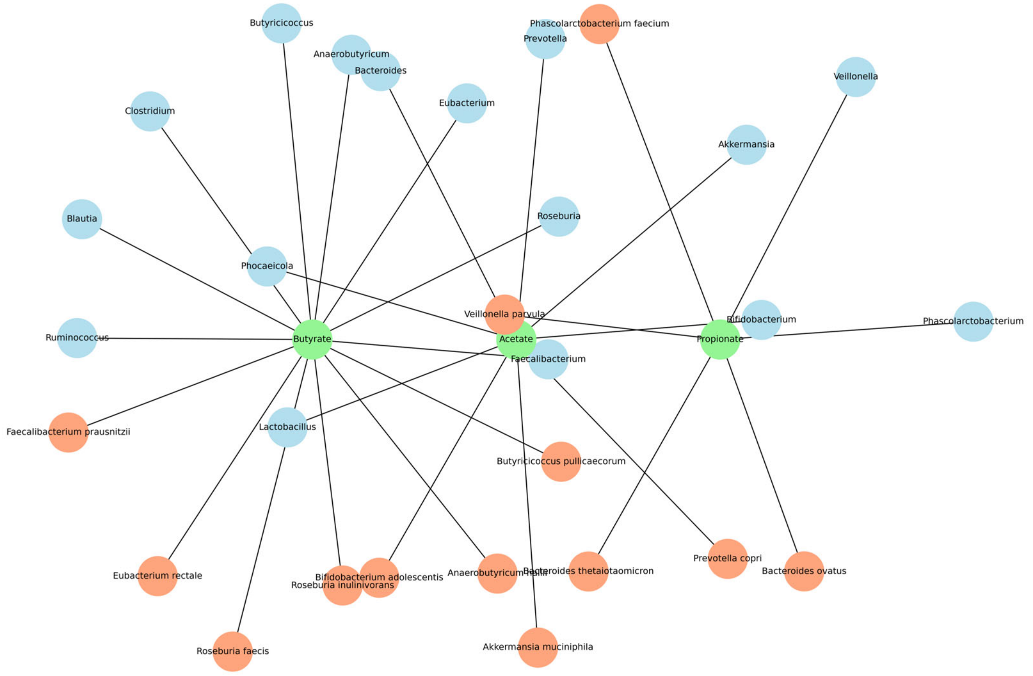

Figure S2. Network diagram of SCFA-producing microbes at the genus and species levels. The diagram illustrates the associations between SCFA types—butyrate, acetate, and propionate (green nodes)—and their corresponding microbial taxa. Microbial genera are shown as blue nodes, while species are represented by orange nodes.

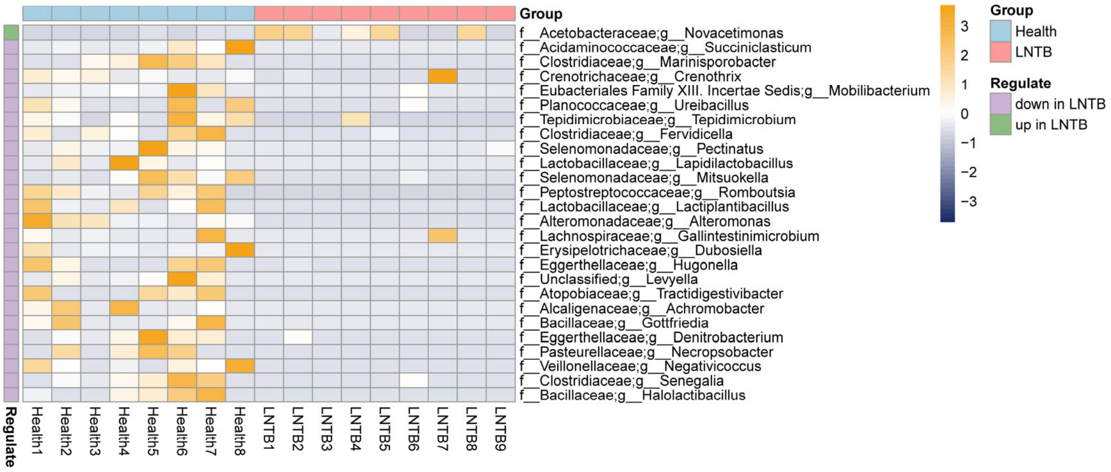

Figure S3. Heatmap of genus-level differential analysis based on fecal metagenomic data from LNTB patients and healthy controls.

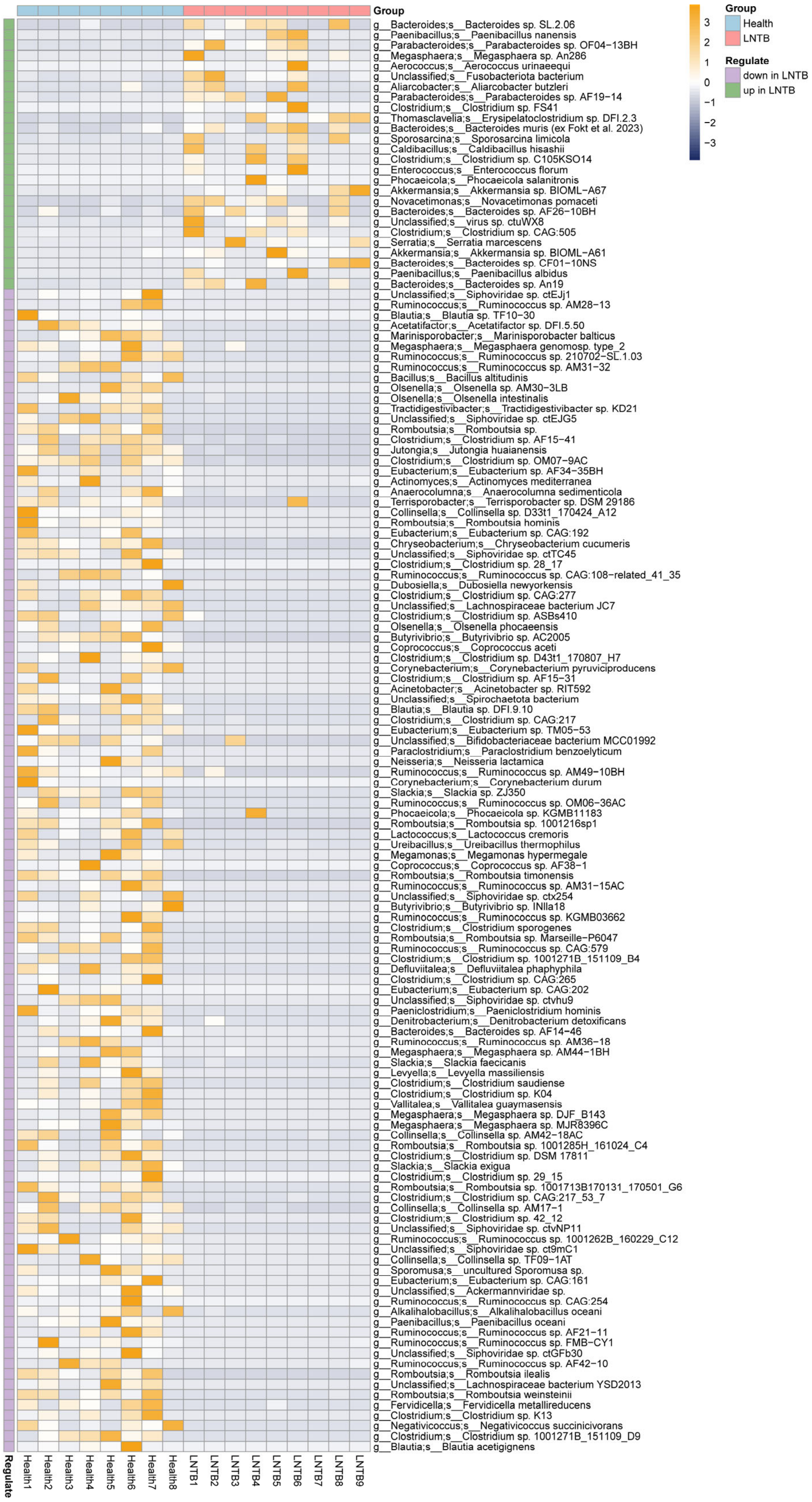

Figure S4. Heatmap of species-level differential analysis based on fecal metagenomic data from LNTB patients and healthy controls.

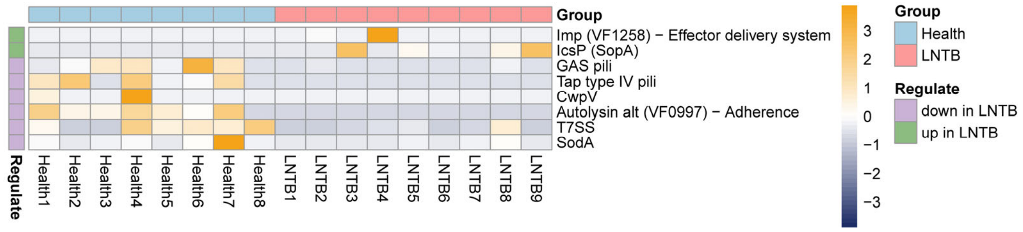

Figure S5. Heatmap of virulence factor profiles at Level 2 based on VFDB annotations from fecal metagenomic data of LNTB patients and healthy controls.

Table S1 Basic Information of LNTB patients

ID	Sex (1=Male; 0=Female)	Age (years)	Height (cm)	Weight (kg)	BMI (kg/m2)
1	0	27	158	56	22.43
2	0	33	156	50	19.43
3	0	35	160	52	20.31
4	1	29	174	71	23.45
5	1	20	170	63.8	22.08
6	0	37	157	54	21.91
7	1	22	177	65	20.75
8	1	35	178	85	26.83
9	0	15	155	49	20.40

Table S2 Diagnosis and Comorbidities of LNTB patients

ID	Diagnosis	Active Pulmonary TB (1=Yes; 0=No)	Old Pulmonary TB (1=Yes; 0=No)	Drug Resistance (1=Yes; 0=No)
1	Right cervical lymph node tuberculosis, abscess type, in regions III, IV, and Vb	0	0	0
2	Recurrent left cervical lymph node tuberculosis post-surgery, abscess type, in regions II, III, IV, V	0	0	0
3	Cervical lymph node tuberculosis, abscess type, in regions IV and Vb	0	0	0
4	Right cervical lymph node tuberculosis, in regions V, IV, III, and VI	0	0	0
5	Cervical lymph node tuberculosis, abscess type, left side, in regions V and IV	0	0	0
6	Left cervical lymph node tuberculosis, abscess type, in regions II and Va	0	0	0
7	Right cervical lymph node tuberculosis, abscess type, in regions II, III, V	0	0	0
8	Cervical lymph node tuberculosis, abscess type, right side, in regions III, IV, V	0	0	0

9	Left cervical lymph node tuberculosis	0	0	0

Table S3 Lymph Node Specimen Pathological Examination Items

ID	Pathological Report	Acid-fast Staining (1=Positive; 0=Negative; ±=Weak Positive)	MTB-PCR (1=Positive; 0=Negative; NA=Not Tested)
1	Granulomatous inflammation with necrosis, acid-fast stain (±)	±	NA
2	Granulomatous inflammation with necrosis, acid-fast stain (±)	1	1
3	Granulomatous inflammation with necrosis, acid-fast stain (±)	0	1
4	Granulomatous inflammation with necrosis, acid-fast stain (±)	±	NA
5	Granulomatous inflammation with caseous necrosis, acid-fast stain (+).	1	NA
6	Granulomatous inflammation with caseous necrosis, acid-fast stain (+).	1	NA
7	Granulomatous inflammation with caseous necrosis, acid-fast stain (+).	1	NA
8	Granulomatous inflammation with necrosis, acid-fast stain (±)	1	NA
9	Granulomatous inflammation with necrosis, acid-fast stain (±)	±	1

Table S4 Blood Specimen Laboratory Tests of LNTB patients

ID	Blood IGRA (1=Positive; 0=Negative; NA=Not Tested)	WBC (10^9/L, Normal: 4.1–11.0)	CRP (mg/L, Normal: 0–6)	ESR (mm/h, Normal: 2–25.7)	PCT (ng/mL, Normal: 0–0.1)
1	1	4	<5.0	27	<0.0200
2	1	4.37	1.96	4	<0.0200
3	NA	6.44	0.61	20	<0.0200
4	NA	6.63	2.54	5	0.038
5	NA	3.3	0.95	13	0.0288
6	1	3.83	<0.6	9	<0.6
7	1	6.5	2.11	9	0.0208
8	1	7.02	11.36	50	0.0549
9	1	5.61	18	<0.6	<0.0200

Table S5 Lymph Node Specimen Examination Items of LNTB patients

ID	Genexpert (1=Positive; 0=Negative; NA=Not Tested)	TB-DNA (1=Positive; 0=Negative; NA=Not Tested)	TB-DNA CT	TB-RNA (1=Positive; 0=Negative)	TB Culture (1=Positive; 0=Negative)
1	NA	1	26.1	0	0
2	NA	1	26.8	0	0
3	NA	1	33.4	1	0
4	1	1	34.5	1	0
5	0	1	33.6	0	0
6	1	1	28.8	0	0
7	1	1	29.2	0	0
8	NA	1	26.5	1	1
9	1	1	31.5	0	0

Table S6 The list of correlation-associated metabolites

ID	Metabolite ID	Full Name	ID	Metabolite ID	Full Name
**1**	Com_10046_neg	PE 20:1	**31**	Com_321_neg	3-(1-pyrrolidinyl)-5-
**2**	Com_1038_pos	Kahweol	**32**	Com_3757_pos	LPC 22:1-SN1
**3**	Com_10712_neg	ST 24:1;O3;S24:1;O3;S	**33**	Com_382_neg	4-Methylphenol
**4**	Com_1221_pos	2-Aminobenzenesulfonic acid	**34**	Com_3963_neg	6α-Prostaglandin I1
**5**	Com_12317_pos	Trenbolone acetate	**35**	Com_4094_pos	Sulfisoxazole
**6**	Com_12527_pos	Lysopc 18:2	**36**	Com_418_pos	diethyl 2-(3-chloroanilino)malonate
**7**	Com_12809_neg	alpha-Benzylsuccinic acid	**37**	Com_4462_neg	23-Nordeoxycholic acid
**8**	Com_12947_pos	Levodopa	**38**	Com_4506_pos	CAR 12:3
**9**	Com_14060_neg	Terephthalic Acid	**39**	Com_4729_pos	2-{2-[(1-methyl-1H-pyrazol-5-yl)amino]-2-oxoethoxy}acetic acid
**10**	Com_141_pos	Indole-3-acetic acid	**40**	Com_49_neg	Phloretin
**11**	Com_1465_neg	o-Cresol	**41**	Com_5262_neg	Methyl 3-indolyacetate
**12**	Com_152_pos	CAR 10:1	**42**	Com_5403_pos	4-Hydroxybenzoic acid
**13**	Com_1635_neg	Phloroglucinol	**43**	Com_542_pos	N'2-acetylpyridine-2-carbohydrazide
**14**	Com_174_neg	Glycoursodeoxycholic acid	**44**	Com_6148_neg	Asaraldehyde
**15**	Com_1856_neg	tetranor-12(R)-HETE	**45**	Com_6163_pos	2-(4-chlorophenyl)-5-(4-phenylbuta-1,3-dienyl)-2H-1,2,3,4-tetraazole
**16**	Com_1954_pos	ethyl 3-oxo-5,6-diphenyl-2,3-dihydropyridazine-4-	**46**	Com_726_neg	ethyl 2-thioxo-4-(trifluoromethyl)-1,2-dihydropyrimidine-5-carboxylate
**17**	Com_2016_neg	Cynaropicrin	**47**	Com_73_pos	1-(2,4-diphenyl-2,3-dihydro-1H-1,5-
**18**	Com_2023_neg	(±)-Abscisic acid	**48**	Com_7585_pos	N-(4-piperidinophenyl)-2-thiophenecarboxamide
**19**	Com_203_pos	Piperine	**49**	Com_781_pos	5-(hydroxymethyl)-4-methoxy-2,5-
**20**	Com_2092_pos	Tolterodine	**50**	Com_8049_pos	3-phenyl-5-(phenylsulfanyl)-1,2,4-
**21**	Com_224_neg	4-tert-Amylphenol	**51**	Com_8070_pos	Taurodeoxycholic Acid (sodium salt)
**22**	Com_2362_pos	SQH	**52**	Com_8084_neg	8-iso-15-keto Prostaglandin F2α
**23**	Com_2448_pos	6-(2-furyl)-2-hydroxy-4-(methylthio)nicotinonitrile	**53**	Com_8295_neg	Estrone
**24**	Com_2569_neg	3-Methylindole	**54**	Com_845_pos	LPC 18:3
**25**	Com_2579_pos	Indoxylsulfuric acid	**55**	Com_860_neg	Indole-3-pyruvic acid
**26**	Com_2702_pos	N-Methyloctan-1-amine	**56**	Com_866_neg	Indole-3-acrylic acid
**27**	Com_2747_pos	L-cysteine	**57**	Com_8717_neg	Prostaglandin E1
**28**	Com_2829_pos	Nicotinuric Acid	**58**	Com_883_neg	Catechin
**29**	Com_3034_pos	Gly-Tyr	**59**	Com_9501_neg	D-Phenylalanine
**30**	Com_317_pos	Hippuric acid	**60**	Com_99_pos	2-({2-[(3-methyl-5-cinnolinyl)amino]-2-oxoethyl}sulfanyl)acetic acid
